# Causes of Complaint against Vaccination

**Published:** 1882-07

**Authors:** Jerome Walker

**Affiliations:** Brooklyn


### CAUSES OF COMPLAINT AGAINST VACCINATION.

Most physicians, who have carefully studied reliable statistics as to the good results of *proper* vaccination, believe that vaccination, with the proper virus, used in the proper way, at the right time, is the only preventive (outside of inoculation) against the ravages of smallpox we have, and which, in an immense *majority* of instances, will completely prevent it.

Unfortunately, many medical students (as I know from conversation with them) have confused ideas, at graduation, as to the extent of the protection which vaccination affords, and are probably led to believe that one vaccination will *always* prevent smallpox. The laity, drawing their information from such physicians, or from some of the public prints, believe it, until they hear of a case of smallpox occurring in a vaccinated person, and then they became anti-vaccinationists; not recognizing the fact that such a case is *very* rare, and that *proper* vaccination, in ninety-nine cases out of a hundred, will completely prevent .smallpox, or greatly lessen its virulence, converting it into varioloid (*i. e.*, modified smallpox), which is rarely or never fatal.

Medical colleges, on the one hand, by failure in instruction of students, and the Society of Anti-Vaccinationists on the other, by misstatements, have given the profession and the people at large causes for complaint as to the *unreliability of information* upon the subject of vaccination. Let the colleges generally see to it that instruction is given in the history of inoculation and vaccination; the respective value and mode of collecting humanized and bovine virus, and when *not* to vaccinate. Boards of health can do great good by circulating in public places, to institutions and schools, circulars giving such information as will tend to assure the laity of the *real* value of vaccination and of the sources of the vaccine virus used.

The Committee on Hygiene of the Medicale Society of the County of Kings, in a report to that Society in September, 1880, stated that the causes of bad or inert vaccinations, as far as could be ascertained, were carelessly conducted vaccination, but especially unreliable virus and virus from unknown sources. The report says: "There are some thirteen physicians and so-called 'purveyors' who advertise to sell virus. There are many who profess to furnish it, but who, when called upon to furnish a quantity, evade direct answers, and ward off a needed supply to 'a more convenient season,' Drug stores sell virus, some from sources which cannot be ascertained." A certain Vaccine Co. (?) was found to consist of one man, with an appendage in the shape of a secretary, who stated to a friend of the investigator that the Co. (?) watched out for the appearance of smallpox in towns and cities, and, when its onset was ascertained, the secretary was sent to the physicians in the places attacked, with *pecuniary* inducements for them to use the Co.'s (?) virus. Then, too, the virus was collected from the calves and *cows* by a workman employed about the place. An examination of some of the crusts furnished for use showed the presence of hairs, dirt and dung. Yet this "vaccine" is largely used!

On inquiry I find that the collection of bovine virus is too often left to other than medical men. After Dr. Martin had introduced bovine virus into this country, and, by a close personal attention to its propagation here, had secured a large annual sale, other men started in the business; at first giving personal attention, then allowing others to propagate and collect for them. This is not as it should be.

The Committee on Hygiene, above referred to, proposed, and the propositions were endorsed by the Society, that hereafter the National Board of Health should supervise the production and dissemination of vaccine virus, especially the bovine.

JEROME WALKER, M.D.

BROOKLYN, February 2, 1882.

